# The predictive validity of a Brain Care Score for dementia and stroke: data from the UK Biobank cohort

**DOI:** 10.3389/fneur.2023.1291020

**Published:** 2023-12-01

**Authors:** Sanjula D. Singh, Tin Oreskovic, Sinclair Carr, Keren Papier, Megan Conroy, Jasper R. Senff, Zeina Chemali, Leidys Gutierrez-Martinez, Livia Parodi, Ernst Mayerhofer, Sandro Marini, Courtney Nunley, Amy Newhouse, An Ouyang, H. Bart Brouwers, Brandon Westover, Cyprien Rivier, Guido Falcone, Virginia Howard, George Howard, Aleksandra Pikula, Sarah Ibrahim, Kevin N. Sheth, Nirupama Yechoor, Ronald M. Lazar, Christopher D. Anderson, Rudolph E. Tanzi, Gregory Fricchione, Thomas Littlejohns, Jonathan Rosand

**Affiliations:** ^1^Henry and Allison McCance Center for Brain Health, Massachusetts General Hospital, Boston, MA, United States; ^2^Nuffield Department of Population Health, University of Oxford, Oxford, United Kingdom; ^3^Department of Neurology, Massachusetts General Hospital, Boston, MA, United States; ^4^Broad Institute of MIT and Harvard, Cambridge, MA, United States; ^5^Center for Genomic Medicine, Massachusetts General Hospital, Boston, MA, United States; ^6^Institute for Health Metrics and Evaluation, University of Washington, Seattle, WA, United States; ^7^Department of Global Health and Population, Harvard T.H. Chan School of Public Health, Boston, MA, United States; ^8^Department of Neurology and Neurosurgery, Brain Center Rudolf Magnus, University Medical Center Utrecht, Utrecht, Netherlands; ^9^Division of Neuropsychiatry, Massachusetts General Hospital, Boston, MA, United States; ^10^Department of Medicine, Massachusetts General Hospital, Boston, MA, United States; ^11^Department of Neurology, Yale School of Medicine, New Haven, CT, United States; ^12^Department of Biostatistics, School of Public Health, University of Alabama at Birmingham, Birmingham, AL, United States; ^13^Department of Medicine (Neurology), University of Toronto, Toronto, ON, Canada; ^14^Krembil Brain Institute, Toronto, ON, Canada; ^15^Lawrence S Bloomberg Faculty of Nursing, University of Toronto, Toronto, ON, Canada; ^16^McKnight Brain Institute, Department of Neurology, School of Medicine, University of Alabama School of Medicine, Birmingham, AL, United States; ^17^Department of Neurology, Brigham and Women’s Hospital, Boston, MA, United States; ^18^Benson-Henry Institute for Mind Body Medicine, Massachusetts General Hospital, Boston, MA, United States

**Keywords:** Brain Care Score, brain health, prevention, risk factors, UK Biobank (UKB), stroke, dementia

## Abstract

**Introduction:**

The 21-point Brain Care Score (BCS) was developed through a modified Delphi process in partnership with practitioners and patients to promote behavior changes and lifestyle choices in order to sustainably reduce the risk of dementia and stroke. We aimed to assess the associations of the BCS with risk of incident dementia and stroke.

**Methods:**

The BCS was derived from the United Kingdom Biobank (UKB) baseline evaluation for participants aged 40–69 years, recruited between 2006–2010. Associations of BCS and risk of subsequent incident dementia and stroke were estimated using Cox proportional hazard regressions, adjusted for sex assigned at birth and stratified by age groups at baseline.

**Results:**

The BCS (median: 12; IQR:11–14) was derived for 398,990 UKB participants (mean age: 57; females: 54%). There were 5,354 incident cases of dementia and 7,259 incident cases of stroke recorded during a median follow-up of 12.5 years. A five-point higher BCS at baseline was associated with a 59% (95%CI: 40-72%) lower risk of dementia among participants aged <50. Among those aged 50–59, the figure was 32% (95%CI: 20-42%) and 8% (95%CI: 2-14%) for those aged >59 years. A five-point higher BCS was associated with a 48% (95%CI: 39-56%) lower risk of stroke among participants aged <50, 52% (95%CI, 47-56%) among those aged 50–59, and 33% (95%CI, 29-37%) among those aged >59.

**Discussion:**

The BCS has clinically relevant and statistically significant associations with risk of dementia and stroke in approximately 0.4 million UK people. Future research includes investigating the feasibility, adaptability and implementation of the BCS for patients and providers worldwide.

## Introduction

The global brain health crisis is now recognized as among the greatest threats to human well-being (1–4). Currently, in the United States of America (USA), one out of seven people suffers from dementia (5), which is expected to be tripled by 2050 (6). A stroke-related death occurs every 4 min in the USA, with 13 million annual deaths attributable to new stroke cases by 2050 (7, 8). Globally, the burden of disease due to dementia and stroke measured by disability-adjusted life years (DALYs) continues to increase (9). Nearly $53 billion was spent on stroke-related costs in the US between 2017 and 2018 (8). In addition, dementia is argued to be more costly than heart disease or cancer (10, 11). In 2021 alone, $352 billion was estimated to have been spent on Alzheimer’s and related dementias (12).

Primary prevention has substantially contributed to lowering mortality rates due to heart disease by 27% and cancer by 32% since 1991 (13). The American Heart Association (AHA) published Life’s Simple Seven (LSS) in 2010 (14) and an updated version, Life’s Essential Eight (LEE) in 2022 (15), aiming to promote health preservation and disease prevention, focusing on heart disease and stroke (cardiovascular disease). These scores were not developed with input from patients, and do not have a focus on brain health (as they excluded dementia). In order to engage patients, we sought to develop a tool that responded to the question we received most frequently from our patients and their family members: “*How can I take good care of my brain?*.” While the development of novel therapies that effectively treat or prevent dementia and stroke remains a top priority, epidemiological studies have consistently identified another powerful opportunity: up to 40% of dementia cases have been attributed to modifiable risk factors, implying that as many are preventable (16). The figure for stroke is even higher, with estimates indicating that at least 60% of stroke cases could be prevented through modification of risk factors (including behavioral changes) (8, 17–19). Importantly, many of the modifiable risk factors for stroke and dementia are shared between these two brain diseases. Despite the potential to substantially reduce the number of new cases of dementia and stroke, achieving primary prevention of brain disease is not yet a part of routine medical care (3).

**Figure 1:**
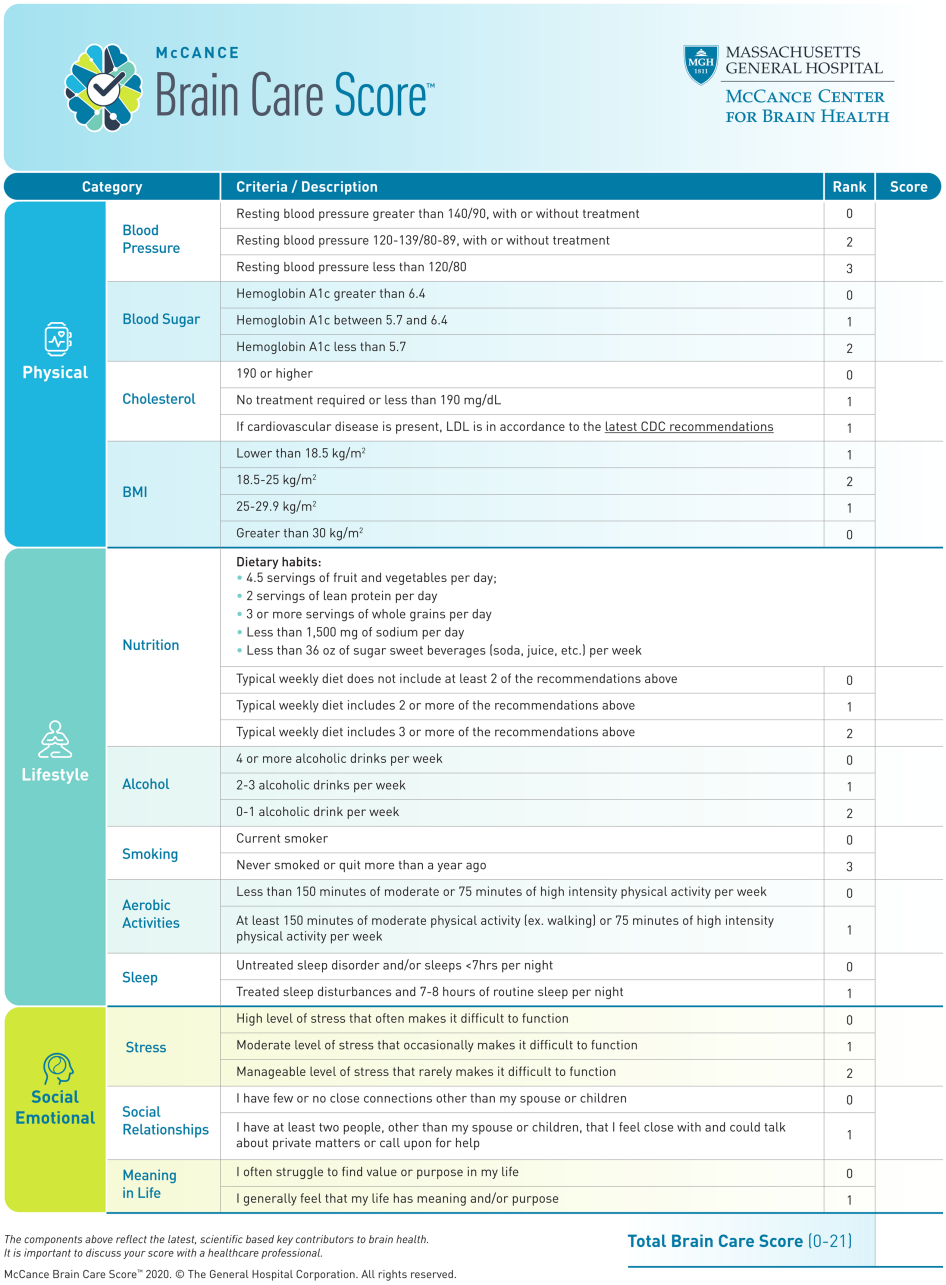
Brain Care Score.

The Brain Care Score (BCS) was developed at the McCance Center for Brain Health in partnership with patients to serve as an evidence-based and pragmatic instrument to engage patients in behavior change toward risk factor modification (20). The BCS represents a larger paradigm change from treating brain disease reactively to actively promoting *brain care*. In contrast to traditional risk scores, which are designed to provide risk stratification for the development of future disease (21–25), the BCS was designed for a different purpose: as a simple tool to motivate patients and their practitioners to modify risk factors and reduce their risk of developing common age-related brain disorders. The BCS ranges from 0–21 and consists of four physical components (blood pressure, hemoglobin A1c, cholesterol, and Body Mass Index [BMI]), five lifestyle elements (nutrition, alcohol intake, smoking, aerobic activities, and sleep), and three social factors (stress, relationships, and purpose in life) (Figure 1).

In the present study, we perform the first validation of the associations of the BCS with incident dementia and stroke using data from the United Kingdom Biobank (UKB), a population-based, prospective cohort study of >500,000 UK participants aged 40–69 recruited between 2006 and 2010. We hypothesized that a higher BCS derived from baseline measurements in the UKB cohort would be associated with a lower incidence of dementia and stroke.

## Methods

### UKB study

The protocol, study design, and inclusion and exclusion criteria of the UKB study have been described elsewhere (26). Briefly, the UKB study is a population-based prospective cohort study of half a million volunteer UK participants who were recruited from 22 centers across the United Kingdom. Detailed data from eligible participants were collected at baseline (2006–2010) through questionnaires and anthropometric and biomedical measurements. The recruitment age was 40–69 years, with only a negligible number of people joining the UKB study who were outside of this age range (mainly through attending the assessment centers with those invited to participate in the UKB study). To date, there have been three follow-up assessments (2012–2013, 2014+, and 2019+). Health outcomes are routinely collected for all participants through linkage to health data, including hospital and mortality data. The UKB study was conducted according to the guidelines laid down in the Declaration of Helsinki and approved by the Northwest Multi-Centre Research Ethics Committee (reference number 06/MRE08/65). Informed consent was obtained from all human research participants of the UKB study.

### Derivation of the BCS

The BCS was designed at the McCance Center for Brain Health. In an effort to maximize practitioner and patient engagement, the BCS was derived through a modified Delphi process (27) that included structured information flow, giving feedback in multiple rounds and, having a facilitator present. For inclusion, we selected common modifiable risk factors for dementia and stroke that were most widely and repeatedly endorsed by professional societies and patient advocacy groups. We then developed a scoring system with weighting scores that was designed to give more emphasis to risk factor modifications hypothesized to be potent in their association with the risk of disease (20). Patient input was obtained through qualitative interviews (28) with 48 patients that addressed information overload, the eliciting of clarifying questions, whether patients felt motivated by the BCS, and whether the BCS made them feel empowered to take care of their brain. Risk factors such as age, genetics, education level, and socio-economic status were not considered for inclusion because we focused on factors that can realistically be modified by the patient or practitioner.

### Exposure: derivation of individual BCS components

The BCS was designed at the McCance Center for Brain Health. The BCS captures (i) physical, (ii) lifestyle, and (iii) social and emotional measures, which are quantified by (i) blood pressure, blood sugar, cholesterol, and Body Mass Index (BMI), (ii) nutrition, alcohol consumption, smoking, aerobic activities, and sleep, and (iii) stress, social relationships, and meaning in life (Figure 1) (3). For the current study, the BCS has been derived from the data recorded in the UKB and therefore corresponds to an adapted definition of the BCS (Table 1; for exact variable definitions of the UKB-derived BCS and differences with the original BCS, please see Supplementary Table S1). All individual BCS components were derived by starting with the exact criterion for each component, only adjusting these definitions if necessary for power considerations or if the data were not available in the UKB. For all derived BCS components which are based on self-reporting, we excluded all participants who indicated “do not know” or “prefer not to answer” in the UKB questionnaires (26, 28).

**Table 1:** Brain Care Score in the UKB.

Category		Criteria/description	Rank
Physical	Blood pressure	Systolic *or* diastolic blood pressure of greater than 140/90 mmHg	0
		Systolic *or* diastolic blood pressure 120–140/80–90 mmHg, and systolic *and* diastolic blood pressure lower than 140/90 mmHg	2
		Systolic *and* diastolic blood pressure lower than 120/90 mmHg	3
	Blood glucose	Hemoglobin A1c greater than 6.4%	0
		Hemoglobin A1c between 5.7 and 6.4%	1
		Hemoglobin A1c less than 5.7%	2
	Cholesterol	Total cholesterol 190 mg/dL or higher	0
		Total cholesterol less than 190 mg/dL	1
	Body mass index	Lower than 18.5 kg/m^2^	1
		Between 18.5 and 25 kg/m^2^	2
		Higher than 25 and lower than 30 kg/m^2^	1
		Higher than or equal to 30 kg/m^2^	0
Lifestyle	Nutrition	Dietary habits: 4.5 or more servings of fruit and vegetables per day A red meat score of 1 or 2 3 or more servings of bread slices or cereal bowls per day Sometimes, rarely, or never add salt to a meal	
		Typical diet does not include at least 2 of the recommendations above	0
		Typical diet includes 2 of the recommendations above	1
		Typical diet includes 3 or more of the recommendations above	3
	Alcohol consumption	Drinking ≥3 times/week	0
		Drinking 1–2 times/week or 1–3 times/month	1
		Drinking only on special occasions or never	2
	Smoking	Current smoker	0
		Former or never smoker	2
	Aerobic activities	At least 10 min of moderate or vigorous activity on fewer than 5 days/week	0
		At least 10 min of moderate or vigorous activity on 5 or more days/week	1
	Sleep	Less than 7 hours/day	0
		7 or more hours/day	1
Social emotional	Stress	Self-perceived tension, fidgetiness, or restlessness several days, more than half the days, or nearly every day in the last 2 weeks	0
		No self-perceived tension, fidgetiness, or restlessness in the last 2 weeks	1
	Social relationships	No friends or family members outside the household; no or almost no visits, or only once every few months	0
		Visits once a month, once a week, two to four times a week, or almost daily	1
Total Brain Care Score (0–19)			

The red meat score is based on beef, pork, and lamb/mutton consumption, in which an individual score was first assigned for each meat type (“Never” or “Less than once a week” with 0; “Once a week” or “2–4 times a week” with 1; and “5–6 times a week” or “Once or more daily” with 2); these were then summed, with a score of 1–2 dichotomized into 1 and less than 1 or more than 2 with a 0. Moderate activity includes physical activities such as carrying light loads or cycling; vigorous activity includes activities such as fast cycling, aerobics, or heavy lifting.

The BCS was calculated as the sum of the assigned scores of all quantified components for each person. In other words: If a person always gets the lowest score in all the components, they have a BCS of 0; if a person always gets the highest score — 1, 2, or 3, depending on the component — they have a BCS of 19. The original BCS ranged from 0 to 21 (Figure 1), but the BCS derived from the UKB ranges from 0 to 19 due to adjustments to the score in nutrition, stress, and meaning of life, as described before. A higher total BCS corresponds with better brain care. A five-point increase in total BCS (e.g., from 0–5 or 10–15) is considered to reflect a substantial yet achievable improvement in someone’s brain care. Hence, an improvement in the BCS of 5 points could be used as an initial goal for patients and providers, with several options for achieving that improvement. Three examples of a 5-point increase in the BCS are: quitting smoking and reducing stress (no tension, fidgetiness, or restlessness in the last 2 weeks) and improving social relationships (visiting family or friends at least once a month); or lowering alcohol consumption (from 4 units/week to <1 unit/week or drinking on special occasions), and lowering blood pressure (from >140/90 mmHg to 120/80 mmHg or comparable); or losing weight (from a BMI ≥30 kg/m^2^ to 18.5–25 kg/m^2^) and lowering blood sugar (from HbA1c >6.4 to HbA1c <5.7).

All information used for quantification of the individual components and subsequently, of the total BCS, was collected at the baseline UKB measurement.

### Outcome assessment: incident dementia and incident stroke

Using the routinely collected hospital and mortality data, the UKB Outcome Adjudication Group, in collaboration with clinical experts, developed algorithms that identify cases of all-cause dementia and stroke. Hospital data linked with the UKB Cohort are from Hospital Episode Statistics for England (censoring date: 30 September 2021), the Scottish Morbidity Record (31 July 2021), and the Patient Episode Database for Wales (28 February 2018); mortality data for England and Wales are provided by NHS Digital (censoring date: 30 September 2021) and the NHS Central Registries, National Records of Scotland (31 October 2021). The full list of codes from the International Classification of Disease, editions 10 and 9 (ICD-10, ICD-9) used in the algorithmic definition of dementia and stroke cases are available at the UK Biobank website. Any primary or secondary diagnosis or a contributory cause of death citing the included ICD codes was considered a case, except for events that occurred before the baseline measurement or in the first 2 years of follow-up (which was defined according to the data source listed above). The exclusion of such events was done to address any concerns of reverse causation (29).

### Statistical analyses

#### Complete case analyses

We included all UKB participants with complete data on the BCS available and excluded all participants who had any missing information on one or more individual BCS components (except ‘meaning of life’ which was missing for all UKB participants). We compared age, sex assigned at birth, and number of incidence stroke or dementia cases between the UKB participants who had complete data on the BCS (included in this study) and those who did not have complete data available (excluded in this study), to detect any potential selection bias.

#### Distribution of the BCS components and the total BCS

We reported the distribution of the measurements, self-reported responses, and any missingness from the UKB questionnaires. In addition, the distribution of the total BCS of included UKB participants was shown, and the median and interquartile range (IQR) or mean and standard deviation (SD) were reported.

#### Cox proportional hazard regression models

To estimate the associations of incident dementia and stroke with five-point differences in the BCS, we employed Cox proportional hazard regression models on non-stratified samples — adjusting for sex assigned at birth (female versus male) and age at baseline (as a continuous variable) — and separately in samples stratified by age group at baseline (<50, 50–59, >59 years) to assess any differences, adjusting for sex assigned at birth. Time to event for the cases was defined as the number of days from the baseline survey to the date of the first occurrence of dementia or stroke. For the other participants, time to event was defined as the number of days to the censoring date, depending on the source of hospital data (listed above), or, for those who died due to other causes, as the date of death. We performed Cox regression models on *outcome 1*: dementia, *outcome 2*: stroke, and *outcome 3*: dementia *or* stroke. When estimating the per 5-point BCS risk difference for dementia *or* stroke as a composite outcome, we used the date of the first outcome that occurred during the follow-up period. The median time to event and follow-up time, along with the interquartile ranges, were reported for all the outcomes. The Cox proportional hazard regression analyses yielded estimated hazard ratios (HR) and corresponding 95% confidence intervals (CI). Schoenfeld residuals were plotted to assess whether the proportional hazards assumption was satisfied. To assess the predictive accuracy of our models, we computed and reported the concordance statistics (c-statistics), which measure the area under the receiver operating characteristic curve. We simulated and visualized the HRs and 95% CI for dementia and stroke risk, for each age group, as a dose–response risk curve over the range of total BCS (0 to 19) using a method by King, Tom, and Wittenberg (30, 31): the mean BCS per group was considered the reference group, and for this, we ran 10,000 simulations per model.

#### Secondary analyses and sensitivity analyses

We performed secondary analyses *(a)* statistically testing the differences in the associations of the BCS with the three outcomes across age and sex assigned at birth strata: *(b)* estimating the absolute risk across quintiles of the BCS, and *(c)* sensitivity analyses assessing a potential bias due to competing risks of death due to other causes.

##### A. Testing the differences in the associations of the BCS across groups (age groups and sex assigned at birth)

Using Cox proportional hazard models featuring two-and three-way interactions between the BCS, age, and sex assigned at birth, we tested any differences between the associations of the BCS across groups as reported in the main analyses.

##### B. Absolute risk estimates across quintiles of the BCS

We provided estimates of the absolute risk and the differences in absolute risk across different ranges of the BCS (in addition to the relative risk estimates from the Cox proportional hazard models). We reported the cumulative incidence and 95% CI in participants with a “low BCS” reflecting suboptimal brain care (defined as 1^st^ quintile); in participants with a “medium BCS” (defined as 2^nd^, 3^rd^, and 4^th^ quintiles) and participants with a “high BCS” reflecting optimal brain care (defined as 5^th^ quintile). To estimate the 95% CI of the cumulative incidence of dementia and stroke for all included participants as well as stratified by age categories, we used the Agresti-Coull method (5).

##### C. Sensitivity analyses: competing risk of death due to other causes

In sensitivity analyses, we used Fine and Gray subdistribution hazard models to estimate the association of the BCS with incident dementia, stroke, and the composite outcome accounting for the competing risk of death due to any other cause (which prevents the outcome of interest from occurring): a substantial difference between estimates from the main analyses and those from the sensitivity analyses would point to a possible bias in the former (32). The Fine and Gray model analyses yielded subdistribution, cause-specific HRs, and 95% CI. These HRs are estimates of the relative difference in the rate of the occurrence of the event of interest (incident dementia, stroke, or both) among subjects who have not yet experienced the event of interest but may have experienced a competing event (30).

All statistical analyses were performed using R 4.2.1 (33). The current manuscript is written in line with the STROBE (Strengthening the Reporting of Observational Studies in Epidemiology) guidelines (Supplementary Table S2).

## Results

### Cohort characteristics

The UKB enrolled 502,408 participants between 2006 and 2010. We performed a complete case analysis and included 79% of all UKB participants by excluded 103,419 participants (21%) due to missing data on one or more of the individual BCS components. A total of 398,900 participants (mean age: 57, of which 54% were females) were included in the final analyses.

When comparing UKB participants with complete data on the BCS (*included in this study*) with UKB participants without complete data on any of the components of the BCS (*excluded from this study*), there were no meaningful differences in age or sex assigned at birth (Supplementary Table S3). Differences were found in incidence of dementia and stroke between UKB participants with and without complete BCS data (Supplementary Table S4).

### Exposure: distribution of BCS components in this cohort

**Figure 2:**
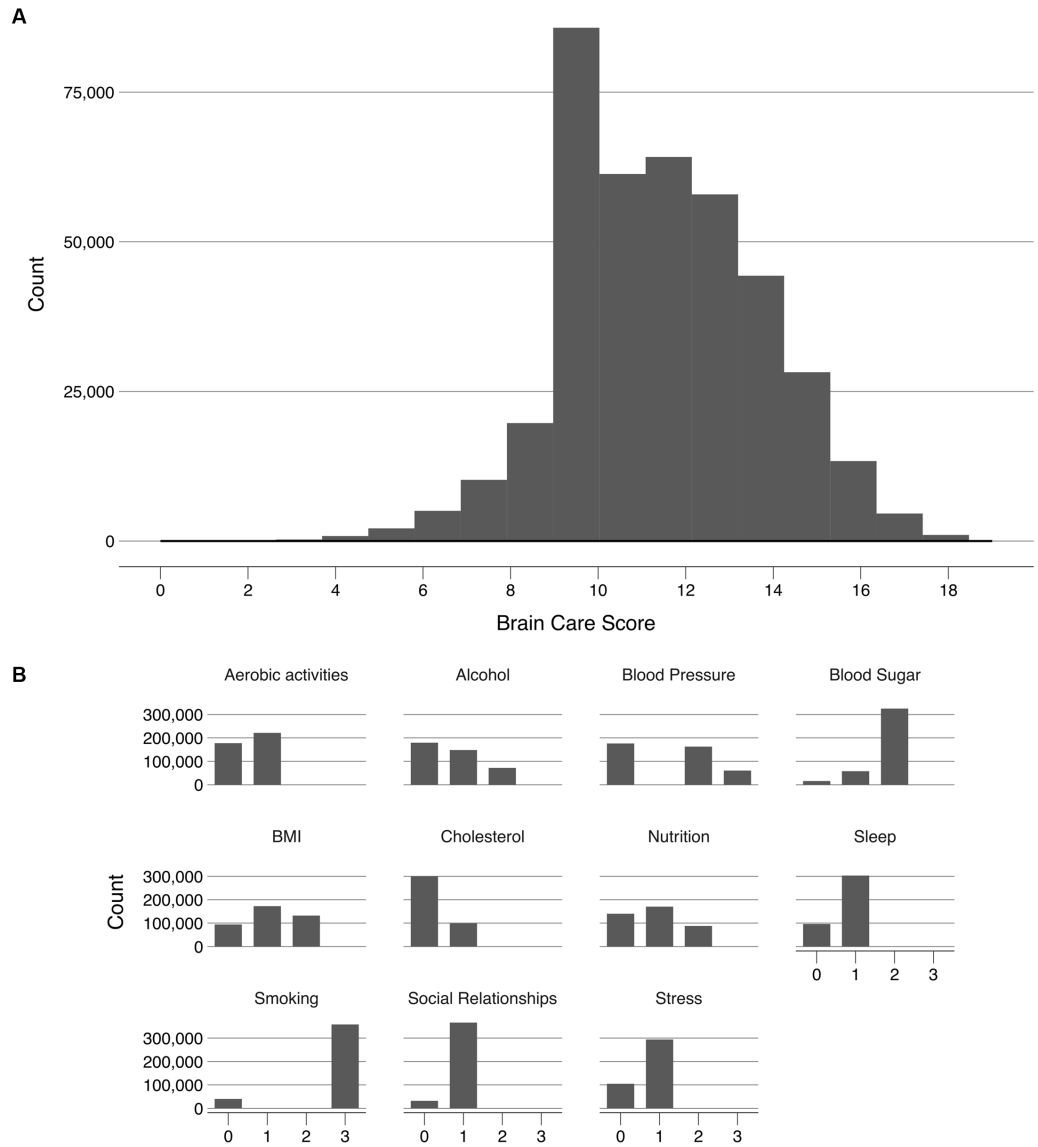
Frequency distribution of the Brain Care Score and its components in the UKB Legend. **(A)** shows the frequency of the total Brain Care Score over the range 1 to 19 observed in participants of the UKB study; **(B)** shows the frequencies of scores from the individual components of the total Brain Care Score.

The distributions of all UKB measurements making up the BCS are shown in Figure 2. Responses from the UKB questionnaires (mean and standard deviations, or frequencies for categorical variables), along with missingness percentage (%), stratified by three age categories (<50, 50–59, >59 years), are provided (Table 2). The missingness for the BCS components at baseline ranged from 0.3% for blood pressure to 7.2% for blood sugar.

**Table 2:** Cohort characteristics at baseline entry into the UKB.

	<50 years (*n* = 117,830)	50–59 years (*n* = 167,115)	>59 years (*n* = 217,463)	Overall (*N* = 502,408)
*Sex assigned at birth*				
Females	64,641 (54.9%)	94,004 (56.3%)	114,679 (52.7%)	273,324 (54.4%)
Males	53,189 (45.1%)	73,111 (43.7%)	102,784 (47.3%)	229,084 (45.6%)
*Age*				
Mean (SD)	45.0 (2.74)	54.8 (2.88)	64.1 (2.85)	56.5 (8.09)
*Systolic blood pressure*				
Mean (SD)	129 (15.9)	136 (17.7)	144 (18.7)	138 (18.7)
Missing	374 (0.3%)	408 (0.2%)	543 (0.2%)	1,325 (0.3%)
*Diastolic blood pressure*				
Mean (SD)	81 (10.4)	82 (10)	83 (10.2)	82 (10.2)
Missing	374 (0.3%)	541 (0.2%)	408 (0.2%)	1,323 (0.3%)
*Glycated hemoglobin (HbA1C) in %*				
Mean (SD)	5.27 (0.547)	5.45 (0.624)	5.56 (0.630)	5.46 (0.620)
Missing	8,713 (7.4%)	12,148 (7.3%)	15,133 (7.0%)	35,994 (7.2%)
*Cholesterol (mg/dL)*				
Mean (SD)	212 (39.4)	225 (43.0)	221 (47.1)	220 (44.3)
Missing	7,844 (6.7%)	10,967 (6.6%)	14,095 (6.5%)	32,906 (6.5%)
*BMI*				
Mean (SD)	27.0 (4.98)	27.5 (4.97)	27.6 (4.55)	27.4 (4.80)
Missing	763 (0.6%)	1,020 (0.6%)	1,321 (0.6%)	3,104 (0.6%)
*Fruit and vegetable servings per day*				
Mean (SD)	3.26 (2.17)	3.60 (2.20)	3.83 (2.24)	3.62 (2.22)
Missing	3,501 (3.0%)	4,806 (2.9%)	7,091 (3.3%)	15,398 (3.1%)
*Bread and cereal servings per day*				
Mean (SD)	2.29 (1.31)	2.34 (1.28)	2.50 (1.26)	2.40 (1.28)
Missing	3,229 (2.7%)	3,927 (2.3%)	4,879 (2.2%)	12,035 (2.4%)
*Red meat score*				
Mean (SD)	0.853 (0.989)	0.922 (1.03)	1.03 (1.06)	0.952 (1.03)
Missing	1,862 (1.6%)	2,131 (1.3%)	3,005 (1.4%)	6,998 (1.4%)
*Salt added to food*				
Always	6,525 (5.5%)	7,942 (4.8%)	9,959 (4.6%)	24,426 (4.9%)
Usually	12,579 (10.7%)	19,105 (11.4%)	26,700 (12.3%)	58,384 (11.6%)
Sometimes	33,458 (28.4%)	47,381 (28.4%)	59,753 (27.5%)	140,592 (28.0%)
Never/rarely	64,932 (55.1%)	92,344 (55.3%)	120,605 (55.5%)	277,881 (55.3%)
Missing	336 (0.3%)	343 (0.2%)	446 (0.2%)	1,125 (0.2%)
*Alcohol intake frequency*				
Daily or almost daily	17,722 (15.0%)	33,423 (20.0%)	50,608 (23.3%)	101,753 (20.3%)
Three or four times a week	27,276 (23.1%)	40,373 (24.2%)	47,773 (22.0%)	115,422 (23.0%)
Once or twice a week	33,956 (28.8%)	43,520 (26.0%)	51,794 (23.8%)	129,270 (25.7%)
One to three times a month	15,965 (13.5%)	18,414 (11.0%)	21,461 (9.9%)	55,840 (11.1%)
Special occasions only	13,208 (11.2%)	18,349 (11.0%)	26,439 (12.2%)	57,996 (11.5%)
Never	9,253 (7.9%)	12,549 (7.5%)	18,825 (8.7%)	40,627 (8.1%)
Missing	450 (0.4%)	487 (0.3%)	563 (0.3%)	1,500 (0.3%)
*Smoking status*				
Current	16,438 (14.0%)	18,623 (11.1%)	17,901 (8.2%)	52,962 (10.5%)
Previous	28,965 (24.6%)	53,884 (32.2%)	90,174 (41.5%)	173,023 (34.4%)
Never	71,805 (60.9%)	93,766 (56.1%)	107,904 (49.6%)	273,475 (54.4%)
Missing	622 (0.5%)	842 (0.5%)	1,484 (0.7%)	2,948 (0.6%)
*Days per week with 10+ minutes of moderate activity*				
Mean (SD)	3.44 (2.30)	3.48 (2.34)	3.84 (2.33)	3.63 (2.33)
Missing	4,991 (4.2%)	8,211 (4.9%)	14,062 (6.5%)	27,264 (5.4%)
*Days per week with 10+ minutes of vigorous activity*				
Mean (SD)	2.04 (1.95)	1.81 (1.95)	1.75 (1.97)	1.84 (1.96)
Missing	4,559 (3.9%)	8,014 (4.8%)	15,000 (6.9%)	27,573 (5.5%)
*Hours of sleep per day*				
Mean (SD)	7.12 (1.07)	7.06 (1.09)	7.24 (1.14)	7.15 (1.11)
Missing	976 (0.8%)	1,375 (0.8%)	1,863 (0.9%)	4,214 (0.8%)
*Number of days with tension, fidgetiness, or restlessness in the last two weeks*				
Nearly every day	3,062 (2.6%)	3,693 (2.2%)	2,585 (1.2%)	9,340 (1.9%)
Several days	29,998 (25.5%)	37,635 (22.5%)	37,798 (17.4%)	105,431 (21.0%)
More than half the days	4,184 (3.6%)	5,004 (3.0%)	4,238 (1.9%)	13,426 (2.7%)
Not at all	75,096 (63.7%)	113,730 (68.1%)	163,193 (75.0%)	352,019 (70.1%)
Missing	5,490 (4.7%)	7,053 (4.2%)	9,649 (4.4%)	22,192 (4.4%)
*Frequency of friends or family visits*				
Almost daily	9,991 (8.5%)	16,680 (10.0%)	31,085 (14.3%)	57,756 (11.5%)
2-4 times a week	30,292 (25.7%)	46,513 (27.8%)	75,206 (34.6%)	152,011 (30.3%)
About once a week	46,005 (39.0%)	60,754 (36.4%)	69,618 (32.0%)	176,377 (35.1%)
About once a month	18,392 (15.6%)	24,558 (14.7%)	23,527 (10.8%)	66,477 (13.2%)
Once every few months or never	11,032 (9.4%)	15,856 (9.5%)	14,969 (6.9%)	41,857 (8.3%)
Missing	2,118 (1.8%)	2,754 (1.6%)	3,058 (1.4%)	7,930 (1.6%)

Blood sugar levels were measured as hemoglobin A1c (HbA1C). The red meat score is based on beef, pork, and lamb/mutton consumption, in which an individual score was first assigned for each meat type (“Never” or “Less than once a week” with 0; “Once a week” or “2–4 times a week” with 1; and “5–6 times a week” or “Once or more daily” with 2); these were then summed, with a score of 1–2 dichotomized into 1 and less than 1 or more than 2 with a 0. Moderate activity includes physical activities such as carrying light loads or cycling; vigorous activity includes activities such as fast cycling, aerobics, or heavy lifting. BMI stands for Body Mass Index.

### Exposure: distribution of total BCS

Of the included 398,990 UK participants, the median total BCS was 12 (total observed range: 1–19); with a median of 12 for participants aged <50 years, 12 for participants aged 50–59 years, and 11 for participants aged >59 years. The BCS was slightly left-skewed (Figure 2).

### Outcome 1: risk of incident dementia

In total, after excluding 237 prevalent cases of dementia that occurred before baseline or in the first 2 years of follow-up, 5,354 incident cases of dementia were recorded (n = 398,753); the cumulative incidence of dementia was 1.3% in the follow-up period (95% confidence interval [CI]: 1.3–1.4; Supplementary Table S4). The median time to event was 9.8 years and the median follow-up time was 12.5 years (Supplementary Tables S5, S6). Among participants aged <50 at baseline (*n* = 94,347), the cumulative incidence was 0.1% (95% CI: 0.1–0.1), with 88 dementia cases in total. Among those aged 50–59 years, 592 cases of incident dementia were recorded (*n* = 133,657) corresponding to a cumulative incidence of 0.4% (95% CI: 0.4–0.5). Among participants aged >59 (*n* = 170,749), the cumulative incidence was 2.7% (95% CI: 2.7–2.8), with 4,676 dementia cases.

**Figure 3:**
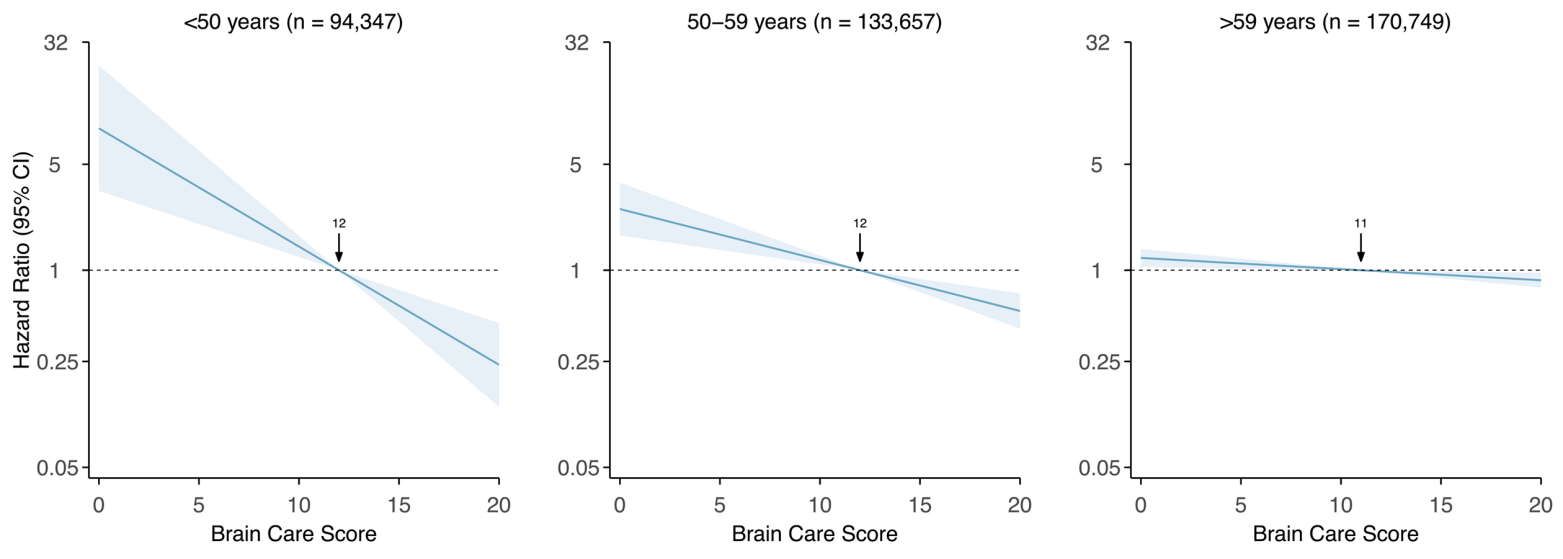
Association of Brain Care Score at baseline with incidence of dementia, stratified by age group at baseline Legend. The thick line is the mean relative hazard curve for dementia incidence over the range of the Brain Care Score on a logarithmic scale; the shaded areas correspond to the 95% confidence intervals. The risk curves were adjusted for sex assigned at birth and plotted relative to the median Brain Care Score (indicated by the arrow) in the respective age group.

A five-point higher BCS was associated with a 14% lower risk of incident dementia when adjusted for age and sex assigned at birth, and this difference was statistically significant [hazard ratio (HR): 0.86 (95% CI: 0.81–0.91), *p*-value: <0.001, c-statistic: 0.81; Figure 3 and Supplementary Table S7]. Among participants aged <50 years at baseline, each five-point higher BCS was associated with a 59% lower risk of incident dementia [HR: 0.41 (95% CI: 0.28–0.60), *p*-value: <0.001, c-statistic: 0.63], adjusted for sex assigned at birth. Among those aged 50 to 59 at baseline, each five-point higher in the BCS was associated with a 32% lower risk of dementia (HR: 0.68 [95% CI: 0.58–0.80], *p*-value: <0.001, c-statistic: 0.58), adjusted for sex assigned at birth. For participants aged >59 years at baseline, each five-point higher BCS was associated with an 8% lower risk of dementia [HR: 0.92 (95% CI: 0.86–0.98), *p*-value: 0.009, c-statistic: 0.54], adjusted for sex assigned at birth (Figure 3).

### Outcome 2: risk of incident stroke

In total, after excluding 6,201 prevalent cases of stroke that occurred before baseline or in the first 2 years of follow-up, 7,259 incident cases of stroke were recorded (*n* = 392,789); the cumulative incidence of stroke was 1.8% (95% CI: 1.8–1.9; Supplementary Table S4). The median time to event was 8.6 years and the median follow-up time was 12.5 years (Supplementary Tables S5, S6). Among participants aged <50 at baseline (*n* = 93,817), the cumulative incidence was 0.6% (95% CI: 0.5–0.6), with 549 stroke cases in total. Among those aged 50–59 years, 1,639 cases of incident stroke were recorded (*n* = 132,086) corresponding to a cumulative incidence of 1.2% (95% CI: 1.2–1.3). Among participants aged >59 (n = 166,886), the cumulative incidence was 3.0% (95% CI: 3.0–3.1), with 5,074 stroke cases.

**Figure 4:**
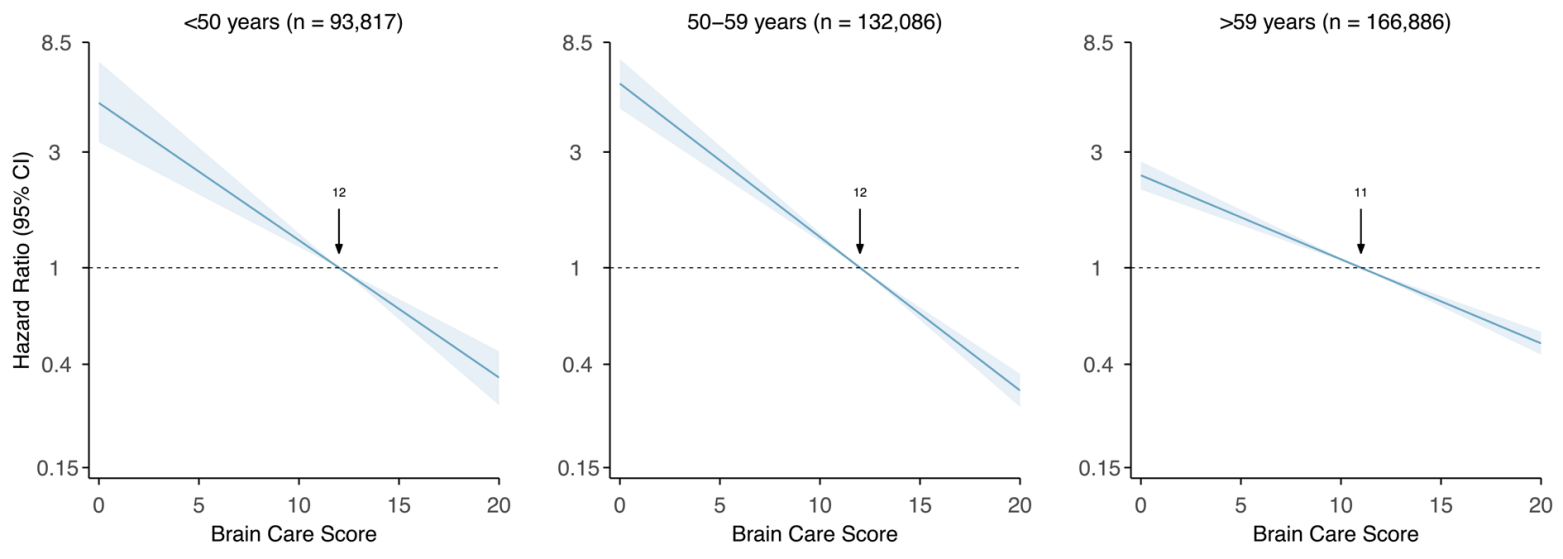
Association of Brain Care Score at baseline with incidence of stroke, stratified by age group at baseline Legend. The thick line is the mean relative hazard curve for stroke incidence over the range of the Brain Care Score on a logarithmic scale; the shaded areas correspond to the 95% confidence intervals. The risk curves were adjusted for sex assigned at birth and plotted relative to the median Brain Care Score (indicated by the arrow) in the respective age group.

Each five-point higher BCS was associated with a 40% lower risk of incident stroke when adjusted for age and sex assigned at birth, and this difference was statistically significant (HR: 0.60 [95% CI: 0.57–0.63], *p*-value: <0.001, c-statistic: 0.71; Figure 4 and Supplementary Table S8). Among participants aged <50 years at baseline, each five-point higher BCS was associated with a 48% lower risk of incident stroke (HR: 0.52 [95% CI: 0.44–0.61], *p*-value: < 0.001, c-statistic: 0.63), adjusted for sex assigned at birth. Among those aged 50 to 59 years at baseline, each five-point higher BCS was associated with a 52% lower risk of stroke (HR: 0.48 [95% CI: 0.44–0.53], *p*-value: <0.001, c-statistic: 0.62), adjusted for sex assigned at birth. For participants aged >59 years at baseline, each five-point higher BCS was associated with a 33% lower risk of stroke (HR: 0.67 [95% CI: 0.63–0.71], *p*-value: <0.001, c-statistic: 0.58), adjusted for sex assigned at birth (Figure 4).

### Outcome 3: combined risk of incident dementia or incident stroke

In total, after excluding 6,416 prevalent cases of dementia *or* stroke that occurred before baseline or in the first 2 years of follow-up 11,710 incident cases of incident dementia *or* stroke were recorded (*n* = 392,574); the cumulative incidence of dementia *or* stroke was 3.0% (95% CI: 2.9–3.0; Supplementary Table S4). The median time to event was 9.1 years and the median follow-up time was 12.5 years (Supplementary Tables S5, S6). Of the 11,710 cases, 586 occurred in participants who experienced both dementia and stroke. For these individuals, the date of the first diagnosis (dementia or stroke) was considered the time of event. Among participants aged <50 at baseline (*n* = 93,817), the cumulative incidence was 0.7% (95% CI: 0.6–0.7), with 632 stroke or dementia cases. Among those aged 50-59 years, 2,115 cases of incident stroke or dementia were recorded (*n* = 132,086) corresponding to a cumulative incidence of 1.6% (95% CI: 1.6–1.7). Among participants aged >59 (*n* = 166,886), the cumulative incidence was 5.3% (95% CI: 5.2–5.5), with 8,923 incident stroke or incident dementia cases.

Each five-point higher BCS was associated with a 30% lower risk of incident stroke *or* incident dementia when adjusted for age and sex assigned at birth, and this difference was statistically significant [HR: 0.70 (95% CI: 0.68–0.73), *p*-value: <0.001, c-statistic: 0.74; Supplementary Table S9]. Among participants aged <50 years at baseline, each five-point higher BCS was associated with a 50% lower risk of incident stroke or dementia [HR: 0.50 (95% CI: 0.43–0.59), *p*-value: < 0.001, c-statistic: 0.62], adjusted for sex assigned at birth. Among those aged 50 to 59 years at baseline, each five-point higher BCS was associated with a 46% lower risk of stroke [HR: 0.54 (95% CI: 0.49–0.58), *p*-value: <0.001, c-statistic: 0.61], adjusted for sex assigned at birth. Finally, for participants aged >59 years at baseline, each five-point higher BCS was associated with a 22% lower risk of stroke [HR: 0.79 (95% CI: 0.75–0.82), *p*-value: <0.001, c-statistic: 0.56], adjusted for sex assigned at birth.

### Secondary analyses and sensitivity analyses

#### A. Testing the differences in the associations of the BCS across groups (age groups and sex assigned at birth)

In secondary analyses statistically testing any differences in associations across the three age groups and sex assigned at birth, two-way interactions between the BCS and age were directionally consistent with the age-stratified primary analyses and statistically significant for some age groups. The interactions of the BCS with sex assigned at birth were close to the null hypothesis (HR of 1) and highly uncertain, as were three-way interactions (Supplementary Table S11).

#### B. Absolute risk estimates across quintiles of BCS

**Figure 5:**
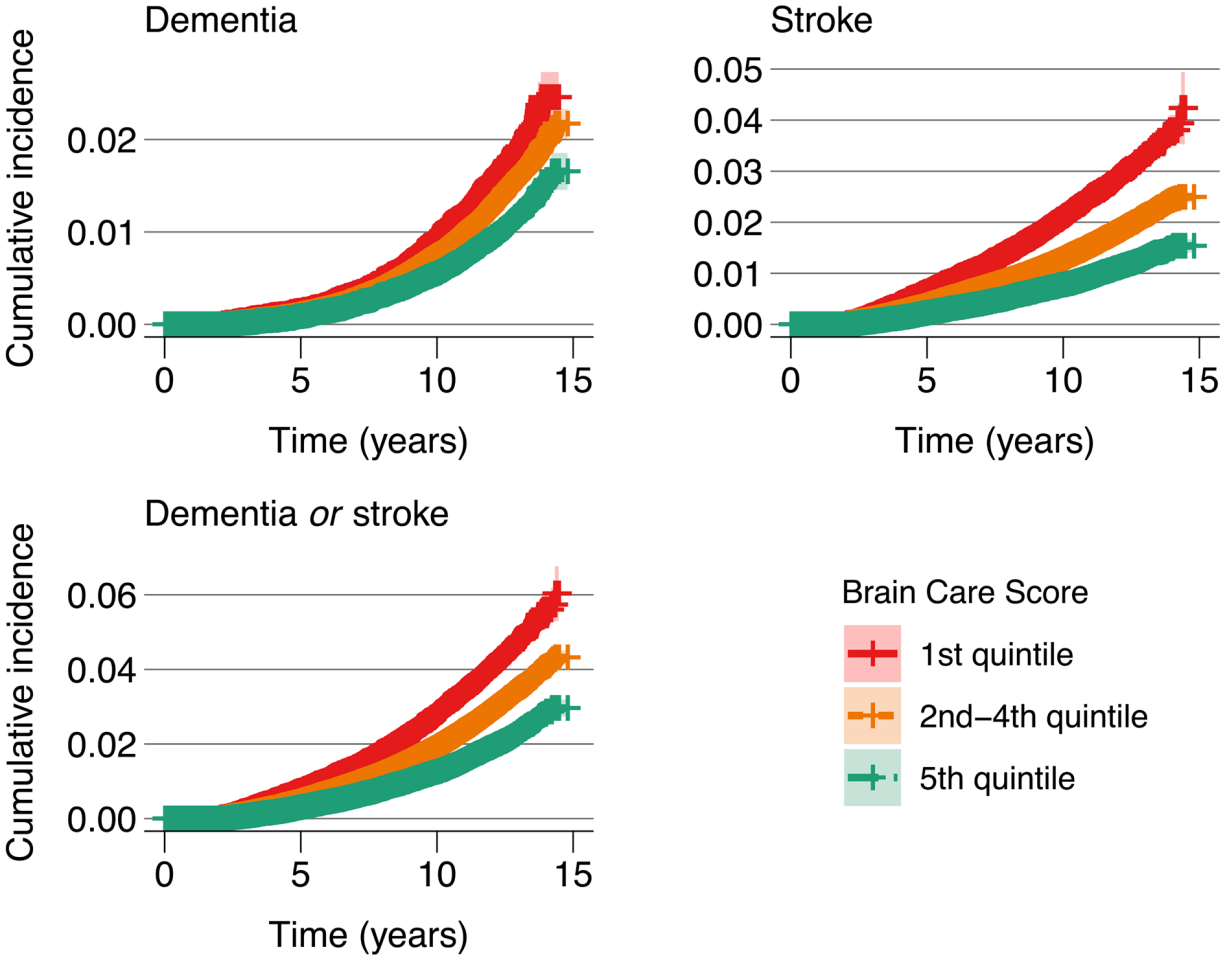
Cumulative incidence of dementia, stroke, and dementia *or* stroke at baseline, stratified by Brain Care Score quintile group Legend. The red line corresponds to the cumulative incidence of the low-scoring BCS group (1st quintile: total BCS scores from 1 to 9), the orange line corresponds to the middle three BCS quintiles (total BCS scores from 10 to 13), and the green line corresponds to the high-scoring BCS group (5th quantile, total BCS scores from 14 to 19).

In a secondary analysis, there was a substantially lower absolute risk of the three outcomes (incident dementia, incident stroke, and incident dementia or stroke) with a higher versus lower total BCS. Individuals were grouped into three categories for analysis: a low-scoring group, with a BCS in the 1st quintile (total BCS scores of 1 to 9); a group with a BCS in the middle three quintiles (with total BCS scores of 10 to 13); and a high-scoring group, with a BCS in the 5th quintile (14 to 19), the highest scores observed in the sample (Supplementary Table S10 for BCS components by quintile groups). The cumulative incidence of all three outcomes was lower in the high-scoring group than in the middle-and — even more substantially — in the low-scoring groups (Figure 5 and Supplementary Table S12).

#### C. Sensitivity analyses: competing risk of death due to other causes and proportional hazards assumption

Based on Fine and Gray subdistribution hazard models, the subdistribution (cause-specific) HR estimates did not differ substantially from estimates from the main Cox regression analyses (Supplementary Tables S13–S15), although the 95% CI of the association between each 5-point higher BCS and risk of dementia among participants aged >59 years included the null. Schoenfeld residuals were plotted in Supplementary Figures S1–S3: no pattern with time is visible for HR estimates, although some of the associated *p*-values indicated statistical significance, which was unsurprising given the sample sizes.

## Discussion

The present study demonstrates clinically relevant and statistically significant associations between the BCS at baseline and incident dementia and incident stroke in nearly 400,000 individuals from the UKB. Higher BCS was associated with a lower risk of dementia and stroke incidence across all age groups available in the UKB (<50, 50–59, >59), with stronger associations reported for younger UKB participants, which may also reflect shorter follow-up for older groups. Sensitivity analyses, consisting of competing risk analyses, provided support for these findings.

The strengths of this study include introducing a first score that focuses on the prevention of both dementia and stroke as common age-related brain diseases which has been developed for ease of interpretation and use by patients and practitioners in primary care. While the BCS may not have optimized components for individual brain diseases, this avoids the creation of separate disease-specific scores. Implementing multiple such scores in routine care would be cumbersome in real-life settings. In fact, a strength of the BCS is that its components are also modifiable risk factors for cardiovascular disease (it includes all the components of AHA Life’s Essential Eight (15)) and for most cancers (34), rendering the BCS an attractive choice if one must settle (a clear, practical benefit) on a single instrument to use routinely in primary care. A further attribute of the BCS is that it does not depend on brain health status at baseline. In other words, the BCS can be used by anyone anywhere, regardless of whether they have a brain disease, have recovered from brain disease, or have never had any brain disease. By avoiding any reference to *brain health,* but rather stimulating *brain care;* the BCS avoids the trap of stigmatization that often accompanies a score that measures the state of one’s (brain) health (35). Although we were limited by the extent of the data collected at baseline for each UKB participant, we were able to construct a very close approximation of the BCS in the UKB (Table 1 and Supplementary Table S1). Nevertheless, the current version of the BCS should be considered merely a prototype tool. Systematic and regular optimization of the BCS is warranted, based on: (i) novel epidemiological evidence between risk factors and brain disease incidence, (ii) systematic reviews and meta-analyses, (iii) observational cohort studies assessing the associations between BCS and late-life depression and/or increases in BCS over time with dementia and stroke incidence, and (iv) investigating the role of genetics (e.g., Apolipoprotein E), (v) motivational aspects of the BCS via mixed-methods research techniques and (vi) cultural-appropriateness of the BCS for different populations via qualitative interviews. A Delphi process, following Delphi research guidelines (36), organized on a yearly basis — taking into consideration all these factors — should assist us in reaching consensus on how the BCS should be optimized.

A current limitation stems from the fact that there is ongoing debate regarding the causal relationship between some of the individual exposures included in the BCS and the outcomes. Particularly for dementia, existing work has encountered multiple challenges in establishing causality between risk factors and dementia incidence (35). Furthermore, for some proposed modifiable risk factors (16), it may be difficult to obtain reliable measurements of the exposure, therefore limiting the possibility of meaningful statistical analyses (37). There is, in addition, suggestive evidence of a difference between the modifiable risk factors for dementia in midlife (40–65 years) and later life (≥65 years) (16, 37, 38). In fact, due to the inclusion criteria of the UKB, we may have missed cases of early-onset dementia, as well as, due to dementia’s long pre-clinical phase, missed milder forms of dementia (39). Our straightforward complete-case analysis included nearly 80% of all UKB participants who did not differ in age or sex assigned at birth from participants who were excluded because of missing data. We did observe a higher incidence of dementia and stroke in excluded participants, which raises the possibility that participants who did not have complete data available on the BCS may have had a lower median BCS at baseline. A larger subsample with repeated BCS measurements (e.g., by using the general practitioner data from the UBK) would have enabled well-powered analyses to quantify associations between within-participant changes in the BCS over time with incidence of dementia and stroke. Because the BCS is designed to be an instrument to be implemented in primary care — and not an epidemiological examination of its constituent components nor a predictive instrument — we refrained from conducting univariable and multivariable analyses between individual BCS components and outcomes or from building more complex, predictive models. We, therefore, did not include any potential confounders of (e.g., statin use for associations between cholesterol and outcomes) or interactions between individual BCS components. Furthermore, we did not conduct an independent analysis of each BCS category to validate the point scales for each. Further iterations and analyses of the BCS within prospective cohort studies and in real-world use will be needed to optimize the number of points allotted for an improvement in each BCS measure.

Primary prevention through risk factor modification and behavior change is crucial if we are to improve global (brain) health, reduce health inequalities, and contain healthcare costs worldwide (3). The US Preventive Services Task Force currently recommends 52 evidence-based preventive services in primary care (40), and professional societies worldwide have generated recommendations for managing modifiable risk factors for heart disease and stroke (38). Nonetheless, achieving measurable improvements in risk factor control (e.g., hypertension) remains challenging (39, 41–47). The BCS has been developed as a tool for use in primary care as a step in overcoming the existing evidence-practice gap for primary prevention of brain disease worldwide (3). Our hope is that the BCS can serve to educate and motivate people to improve their brain care, and consequently reduce the number of new cases of dementia and stroke and delay the onset of these diseases. The present study is the essential first step towards determining whether the BCS can fulfil this promise.

## Data availability statement

The data used in this study can be accessed by contacting the UK Biobank (www.ukbiobank.ac.uk) This analysis was approved by the UK Biobank access committee as part of project 41115.

## Ethics statement

The studies involving humans were approved by Northwest Multi-Centre Research Ethics Committee (reference number 06/MRE08/65). The studies were conducted in accordance with the local legislation and institutional requirements. The participants provided their written informed consent to participate in this study.

## Author contributions

SS: Methodology, Supervision, Writing – original draft. TO: Data curation, Software, Methodology, Writing – original draft. SC: Data curation, Software, Methodology, Writing – original draft. KP: Writing – review & editing. MC: Writing – review & editing. JS: Writing – review & editing. ZC: Conceptualization, Writing – review & editing. LG-M: Conceptualization, Writing – review & editing. LP: Writing – review & editing. EM: Writing – review & editing. SM: Writing – review & editing. CN: Writing – review & editing. AN: Conceptualization, Writing – review & editing. AO: Writing – review & editing. HB: Writing – review & editing. BW: Writing – review & editing. CR: Writing – review & editing. GuF: Writing – review & editing. VH: Writing – review & editing. GH: Writing – review & editing. AP: Writing – review & editing. SI: Writing – review & editing. KS: Writing – review & editing. NY: Writing – review & editing. RL: Writing – review & editing. CA: Conceptualization, Writing – review & editing. RT: Writing – review & editing. GrF: Writing – review & editing. TL: Writing – review & editing. JR: Writing – original draft.

## Supplementary material

The Supplementary material for this article can be found online at:

https://www.frontiersin.org/articles/10.3389/fneur.2023.1291020/full#supplementary-material
