# Corrigendum: The predictive validity of a Brain Care Score for dementia and stroke: data from the UK Biobank cohort

**DOI:** 10.3389/fneur.2024.1501771

**Published:** 2024-11-05

**Authors:** Sanjula D. Singh, Tin Oreskovic, Sinclair Carr, Keren Papier, Megan Conroy, Jasper R. Senff, Zeina Chemali, Leidys Gutierrez-Martinez, Livia Parodi, Ernst Mayerhofer, Sandro Marini, Courtney Nunley, Amy Newhouse, An Ouyang, H. Bart Brouwers, Brandon Westover, Cyprien Rivier, Guido Falcone, Virginia Howard, George Howard, Aleksandra Pikula, Sarah Ibrahim, Kevin N. Sheth, Nirupama Yechoor, Ronald M. Lazar, Christopher D. Anderson, Rudolph E. Tanzi, Gregory Fricchione, Thomas Littlejohns, Jonathan Rosand

**Affiliations:** ^1^Henry and Allison McCance Center for Brain Health, Massachusetts General Hospital, Boston, MA, United States; ^2^Nuffield Department of Population Health, University of Oxford, Oxford, United Kingdom; ^3^Department of Neurology, Massachusetts General Hospital, Boston, MA, United States; ^4^Broad Institute of MIT and Harvard, Cambridge, MA, United States; ^5^Center for Genomic Medicine, Massachusetts General Hospital, Boston, MA, United States; ^6^Institute for Health Metrics and Evaluation, University of Washington, Seattle, WA, United States; ^7^Department of Global Health and Population, Harvard T.H. Chan School of Public Health, Boston, MA, United States; ^8^Department of Neurology and Neurosurgery, Brain Center Rudolf Magnus, University Medical Center Utrecht, Utrecht, Netherlands; ^9^Division of Neuropsychiatry, Massachusetts General Hospital, Boston, MA, United States; ^10^Department of Medicine, Massachusetts General Hospital, Boston, MA, United States; ^11^Department of Neurology, Yale School of Medicine, New Haven, CT, United States; ^12^Department of Biostatistics, School of Public Health, University of Alabama at Birmingham, Birmingham, AL, United States; ^13^Department of Medicine (Neurology), University of Toronto, Toronto, ON, Canada; ^14^Krembil Brain Institute, Toronto, ON, Canada; ^15^Lawrence S Bloomberg Faculty of Nursing, University of Toronto, Toronto, ON, Canada; ^16^McKnight Brain Institute, Department of Neurology, School of Medicine, University of Alabama School of Medicine, Birmingham, AL, United States; ^17^Department of Neurology, Brigham and Women's Hospital, Boston, MA, United States; ^18^Benson-Henry Institute for Mind Body Medicine, Massachusetts General Hospital, Boston, MA, United States

**Keywords:** Brain Care Score, brain health, prevention, risk factors, UK Biobank (UKB), stroke, dementia

In the published article Dr. Westover's disclosure statement was inadvertently omitted. The disclosure should read:

“BW has private equity as co-founder of Beacon Biosignals and receives compensation for consulting and scientific advisory roles.

The remaining authors declare that the research was conducted in the absence of any commercial or financial relationships that could be construed as a potential conflict of interest.”

The authors apologize for this omission and state that this does not change the scientific conclusions of the article in any way. The original article has been updated.

