# Supplementary material for: The predictive validity of a Brain Care Score for dementia and stroke: data from the UK Biobank cohort
Source: Front Neurol. 2023 Dec 1;14:1291020. doi: 10.3389/fneur.2023.1291020 (PMC10725202; doi:10.3389/fneur.2023.1291020)
Supplement: Supplementary file 1 [file Data_Sheet_1.docx]

Supplementary Material

# Title: A Brain Care Score for Dementia and Stroke: data from the UK Biobank cohort

**Sanjula D Singh**†**, Tin Oreskovic**†**, Sinclair Carr**†**, Keren Papier, Megan Conroy, Jasper R Senff, Zeina Chemali, Leidys Gutierrez-Martinez, Livia Parodi, Ernst Mayerhofer, Sandro Marini, Courtney Nunley, Amy Newhouse, An Ouyang, H Bart Brouwers, Brandon Westover, Cyprien Rivier, Guido Falcone, Virginia Howard, George Howard, Aleksandra Pikula, Sarah Ibrahim, Kevin N Sheth, Nirupama Yechoor, Ronald M Lazar, Christopher D Anderson, Rudolph E Tanzi, Gregory Fricchione, Thomas Littlejohns, Jonathan Rosand***

† Equal contribution and first authorship

***Correspondence:**

Corresponding Author: Jonathan Rosand MD MSc 
Email: [jrosand@mgh.harvard.edu](mailto:jrosand@mgh.harvard.edu)
Henry and Allison McCance Center for Brain Health, 399 Revolution Drive, Suite 1160, Somerville, MA 02145, United States

Table of Contents

[Table S1. Differences between the BCS and UKB derived BCS 2](#_Toc140081840)

[Table S2. STROBE Statement 5](#_Toc140081841)

[Table S3. Sample characteristics at baseline among participants with and without complete Brain Care Score data 7](#_Toc140081842)

[Table S4. Cumulative incidence of dementia and stroke at baseline in participants with and without complete Brain Care Score data 7](#_Toc140081843)

[Table S5. Median follow-up time in years for dementia, stroke, and composite outcome analyses 7](#_Toc140081844)

[Table S6. Median time to event in years for dementia, stroke, and composite outcome analyses 7](#_Toc140081845)

[Table S7. Coefficient table for dementia models 8](#_Toc140081846)

[Table S8. Coefficient table for stroke models 8](#_Toc140081847)

[Table S10. Brain Care Score components, by quintile group 9](#_Toc140081848)

[Table S11. Hazard Ratios (95%CI) between the Brain Care Score and risk of dementia, stroke, and dementia *or* stroke 11](#_Toc140081849)

[Table S12. Cumulative incidence of dementia, stroke, and dementia or stroke at baseline by Brain Care Score quintile group 11](#_Toc140081850)

[Table S13. Coefficient table for dementia Fine and Gray subdistribution hazard model 12](#_Toc140081851)

[Table S14. Coefficient table for stroke Fine and Gray subdistribution hazard model 12](#_Toc140081852)

[Table S15. Coefficient table for composite Fine and Gray subdistribution hazard model 12](#_Toc140081853)

[Fig. S1 Schoenfeld residuals for proportional hazards assumption with incident dementia as outcome 13](#_Toc140081854)

[Fig. S2 Schoenfeld residuals for proportional hazards assumption with incident stroke as outcome 15](#_Toc140081855)

[Fig. S3 Schoenfeld residuals for proportional hazards assumption with incident dementia *or* stroke as outcome 17](#_Toc140081856)

| Table S1. Differences between the BCS and UKB derived BCS | |
| --- | --- |
| **Measurement** | **Information** |
| **Blood Pressure** |  |
| Differences BCS and UKB-BCS | None |
| UKB data to score conversion | The mean of two automated, or, if not available, manual blood pressure measurements was calculated and used to score blood pressure |
| **Blood Glucose** |  |
| Differences BCS and UKB-BCS | None |
| UKB data to score conversion | UKB data in mmol/mol was converted to % using the formula: AIC(%) = (A1C(mmol/mol)/10.929)+2.15 |
| **Cholesterol** |  |
| Differences BCS and UKB-BCS | None |
| UKB data to score conversion | UKB data in mmol/L was converted to mg/dL using the formula:  mmol/L*38.66976 |
| **Body Mass Index** |  |
| Differences BCS and UKB-BCS | None |
| UKB data to score conversion | None |
| **Nutrition** |  |
| Differences BCS and UKB-BCS | - 2 servings of lean protein per day was translated into 1–4 servings of red meat per week - 3 or more servings of whole grains per day was translated into 3 or more servings of bread slices or cereal bowls per day - Less than 1500mg of sodium per day was translated into sometimes, rarely or never added salt to a meal - Less than 36 0z. of sugar sweet beverages (soda, juice, etc.) per week was not included as it was not collected in the baseline assessment of the UKB - Alternation was necessary to avoid significant power loss - Self-reports were used |
| UKB data to score conversion …vegetables and fruit | - To convert the reported intake of cooked and salad/raw vegetables from heaped tablespoons to standard serving sizes, we used the American Heart Association's (AHA) Serving Sizes guidelines (42) - UKB-data heaped tablespoons were translated into 2 tablespoons - 8 heaped tablespoons or 16 tablespoons are equivalent to 1 cup - 1 serving of cooked vegetables consists of 4 heated tablespoons equivalent to ½ cup - 1 serving of salad/raw vegetables consists of 8 heaped tablespoons equivalent to 1 cup - 1 serving of fruit consisted of 2 dried fruit portions - At least 4.5 half servings, as stated above, were considered a dietary recommendation^23^ |
| UKB data to score conversion …red meat | - Beef, pork, and lamb/mutton consumption was combined to a red meat score, in which an individual score was first assigned for each meat type and later combined to a total number of red meat servings per week - “Never” or “less than once a week” for each type of red meat were calculated as 0 points - “Once a week” or “2–4 times per week” for each type of red meat were calculated as 1 point - “5–6 times a week” or “Once or more daily” for each type of red meat were calculated as 2 points - A total score of 1–2 points was considered a dietary recommendation, a score of 0 or more than 2 was not |
| **Alcohol consumption** |  |
| Differences BCS and UKB-BCS | - 4 or more alcoholic drinks per week was translated into 3 or more times of alcohol consumption per week - 2–3 alcoholic drinks per week was translated into 1–2 times of alcohol consumption per week or 1–3 times of alcohol consumption per month - 0–1 alcoholic drink per week was translated into never/ special occasions only |
| UKB data to score conversion | - 3 or more times of alcohol consumption per week is 0 points - 1–2 times per week or 1–3 times per month is 1 point - “Never” or “only on special occasions” is 2 points - Alternation was necessary to avoid significant power loss - Self-reports were used |
| **Smoking** |  |
| Differences BCS and UKB-BCS | Never smoked or quit more than a year ago was translated into non-smoker |
| UKB data to score conversion | - Non-smokers are a combination of former smokers and never having smoked - Alternation was necessary, given the lack of data on the time of stopped smoking, to avoid significant power loss - Self-reports were used |
| **Aerobic activities** |  |
| Differences BCS and UKB-BCS | - Less than 150 minutes of moderate or 75 minutes of high intensity physical activity per week was translated into at least 10 minutes of moderate or vigorous activity for less than 5 days per week - At least 150 minutes of moderate physical activity (ex. walking) or 75 minutes of high intensity physical activity per week was translated into at least 10 minutes of moderate or vigorous activity for at least 5 days per week |
| UKB data to score conversion | - At least 10 minutes of moderate or vigorous activity for less than 5 days per week is 0 points - At least 10 minutes of moderate of vigorous activity for at least 5 days per week is 1 point - Moderate activity includes physical activities such as carrying light loads or cycling; vigorous activity includes activities such as fast cycling, aerobics, or heavy lifting - Self-reports were used |
| **Sleep** |  |
| Differences BCS and UKB-BCS | - Untreated sleep disorder and/or sleep <7 hours per night was translated into at less than 7 hours of sleep per day - Treated sleep disturbances and 7–8 hours of routine sleep per night was translated into at least 7 hours of sleep per day |
| UKB data to score conversion | - Less than 7 hours per day of sleep is 0 points - At least 7 hours per day of sleep is 1 point - Data on sleep disorders (treated or untreated) was not available at baseline measurement of the UKB - Self-reports were used |
| **Stress** |  |
| Differences BCS and UKB-BCS | - Moderate level of stress that occasionally makes it difficult to function and high level of stress that often makes it difficult to function were both translated into the presence of self-perceived tension, fidgetiness or restlessness in the last 2 weeks - Manageable level of stress that rarely makes it difficult to function was translated into the absence of self-perceived tension, fidgetiness or restlessness in the last 2 weeks |
| UKB data to score conversion | - The presence of self-perceived tension, fidgetiness or restlessness in the last two weeks is 0 points - The absence of self-perceived tension, fidgetiness or restlessness in the last two weeks is 1 point - As the definition used in the UKB-BCS did not completely match the BCS we scored 0–1, instead of 0–2 - Self-reports were used |
| **Social relationship** |  |
| Differences BCS and UKB-BCS | - I have few or no close connections other than my spouse or children was translated into visits from family or friends outside household less than once a month - I have at least two people, other than my spouse or children, that I feel close with and could talk about private matters or call upon for help was translated into visits from family or friends outside household at least once a month |
| UKB data to score conversion | - The social relationship component was captured by the frequency of friends or family visits - No friends or family members outside the household, no or almost no visits, or only once every few months is a score of 0 points - Visits once a month, once a week, two to four times a week, or almost daily are a score of 1 point - Self-reports were used |
| **Meaning in life** | Due to a lack of information on the self-reported meaning of life in the baseline measurements of the UKB study, we were unable to quantify a corresponding factor and therefore did not include this component |
| **Legend.** BCS = original Brain Care Score, UKB = United Kingdom Biobank, UKB-BCS = United Kingdom Biobank derived Brain Care Score, HbA1c = haemoglobin A1c. | |

| Table S2. STROBE Statement | | | |
| --- | --- | --- | --- |
|  | Item No | Recommendation | Page No |
| **Title and abstract** | 1 | (*a*) Indicate the study’s design with a commonly used term in the title or the abstract | 1 |
|  |  | (*b*) Provide in the abstract an informative and balanced summary of what was done and what was found | 2 |
| Introduction | | | |
| Background/rationale | 2 | Explain the scientific background and rationale for the investigation being reported | 5 |
| Objectives | 3 | State specific objectives, including any prespecified hypotheses | 6 |
| Methods | | | |
| Study design | 4 | Present key elements of study design early in the paper | 7 |
| Setting | 5 | Describe the setting, locations, and relevant dates, including periods of recruitment, exposure, follow-up, and data collection | 7 |
| Participants | 6 | (*a*) Give the eligibility criteria, and the sources and methods of selection of participants. Describe methods of follow-up | 7, 8 |
|  |  | (*b*) For matched studies, give matching criteria and number of exposed and unexposed | N/A |
| Variables | 7 | Clearly define all outcomes, exposures, predictors, potential confounders, and effect modifiers. Give diagnostic criteria, if applicable | 7, 8; Tables 1 and S1 |
| Data sources/ measurement | 8* | For each variable of interest, give sources of data and details of methods of assessment (measurement). Describe comparability of assessment methods if there is more than one group | 7, 8; Table S1 |
| Bias | 9 | Describe any efforts to address potential sources of bias | 8–10 |
| Study size | 10 | Explain how the study size was arrived at | 8 |
| Quantitative variables | 11 | Explain how quantitative variables were handled in the analyses. If applicable, describe which groupings were chosen and why | 8–10 |
| Statistical methods | 12 | (*a*) Describe all statistical methods, including those used to control for confounding | 8–10 |
|  |  | (*b*) Describe any methods used to examine subgroups and interactions |  |
|  |  | (*c*) Explain how missing data were addressed |  |
|  |  | (*d*) If applicable, explain how loss to follow-up was addressed |  |
|  |  | (*e*) Describe any sensitivity analyses |  |
| Results | | |  |
| Participants | 13* | (a) Report numbers of individuals at each stage of study—eg numbers potentially eligible, examined for eligibility, confirmed eligible, included in the study, completing follow-up, and analysed | 11,  Table 2 |
|  |  | (b) Give reasons for non-participation at each stage | N/A |
|  |  | (c) Consider use of a flow diagram |  |
| Descriptive data | 14* | (a) Give characteristics of study participants (eg demographic, clinical, social) and information on exposures and potential confounders | 11,  Tables 2, S3–4, and S10; Figure 2 |
|  |  | (b) Indicate number of participants with missing data for each variable of interest | Table 2 |
|  |  | (c) Summarise follow-up time (eg, average and total amount) | 11–13; Tables S5 and S6 |
| Outcome data | 15* | Report numbers of outcome events or summary measures over time | 11–13; Tables S4 and S12; Figure 5 |
| Main results | 16 | (*a*) Give unadjusted estimates and, if applicable, confounder-adjusted estimates and their precision (eg, 95% confidence interval). Make clear which confounders were adjusted for and why they were included | 11–13; Tables S7–9, S11, S13–15; Figures 3 and 4 |
|  |  | (*b*) Report category boundaries when continuous variables were categorized | 13, 14 |
|  |  | (*c*) If relevant, consider translating estimates of relative risk into absolute risk for a meaningful time period | 11–14; Tables S4 and S12; Figure 5 |
| Other analyses | 17 | Report other analyses done—eg analyses of subgroups and interactions, and sensitivity analyses | 13, 14; Tables S10–15 |
| **Discussion** | | | |
| Key results | 18 | Summarise key results with reference to study objectives | 15 |
| Limitations | 19 | Discuss limitations of the study, taking into account sources of potential bias or imprecision. Discuss both direction and magnitude of any potential bias | 15, 16 |
| Interpretation | 20 | Give a cautious overall interpretation of results considering objectives, limitations, multiplicity of analyses, results from similar studies, and other relevant evidence | 15, 16 |
| Generalisability | 21 | Discuss the generalisability (external validity) of the study results | 15, 16 |
| **Other information** | | | |
| Funding | 22 | Give the source of funding and the role of the funders for the present study and, if applicable, for the original study on which the present article is based | 19 |

| Table S3. Sample characteristics at baseline among participants with and without complete Brain Care Score data | | |
| --- | --- | --- |
|  | **Complete BCS data** | **Incomplete BCS data** |
|  | % (95% CI) | |
| Males | 46% (46% to 46%) | 44% (44% to 45%) |
|  | Mean (95% CI) | |
| Age | 56.45 (56.42 to 56.47) | 56.85 (56.8 to 56.9) |

| **Legend.** 95% CI = 95% confidence interval. |
| --- |

| Table S4. Cumulative incidence of dementia and stroke at baseline in participants with and without complete Brain Care Score data | | | | |
| --- | --- | --- | --- | --- |
|  | **Cumulative incidence of dementia (95% CI)** | | **Cumulative incidence of stroke (95% CI)** | |
|  | **Complete BCS data** | **Incomplete BCS data** | **Complete BCS data** | **Incomplete BCS data** |
| Overall | 1.3% (1.3% to 1.4%) | 2.1% (2.0% to 2.2%) | 1.8% (1.8% to 1.9%) | 2.2% (2.1% to 2.3%) |
| <50 years | 0.1% (0.1% to 0.1%) | 0.2% (0.2% to 0.3%) | 0.6% (0.5% to 0.6%) | 3.5% (3.3% to 3.6%) |
| 50–59 years | 0.4% (0.4% to 0.5%) | 0.7% (0.6% to 0.8%) | 1.2% (1.2% to 1.3%) | 1.6% (1.4% to 1.7%) |
| >59 years | 2.7% (2.7% to 2.8%) | 4.1% (3.9% to 4.2%) | 3.0% (3.0% to 3.1%) | 0.7% (0.6% to 0.9%) |

**Legend.** 95% CI = 95% confidence interval.

| Table S5. Median follow-up time in years for dementia, stroke, and composite outcome analyses | | | |
| --- | --- | --- | --- |
|  | **Median follow-up time for dementia analysis (IQR)** | **Median follow-up time for stroke analysis (IQR)** | **Median follow-up time for the composite outcome (IQR)** |
| Overall | 12.52 (11.75 to 13.21) | 12.52 (11.79 to 13.21) | 12.53 (11.8 to 13.21) |
| <50 years | 12.60 (11.90 to 13.27) | 12.60 (11.90 to 13.27) | 12.60 (11.90 to 13.27) |
| 50–59 years | 12.57 (11.85 to 13.25) | 12.58 (11.85 to 13.25) | 12.58 (11.85 to 13.25) |
| >59 years | 12.40 (11.61 to 13.13) | 12.40 (11.61 to 13.13) | 12.41 (11.63 to 13.14) |

| **Legend.** IQR = interquartile range. |
| --- |

| Table S6. Median time to event in years for dementia, stroke, and composite outcome analyses | | | |
| --- | --- | --- | --- |
|  | **Median follow-up time to dementia diagnosis (IQR)** | **Median follow-up time to stroke diagnosis (IQR)** | **Median follow-up time to event for the composite outcome (IQR)** |
| Overall | 9.84 (7.72 to 11.48) | 8.55 (5.64 to 10.79) | 9.10 (6.38 to 11.07) |
| <50 years | 9.95 (8.32 to 11.40) | 8.32 (5.57 to 10.73) | 8.58 (5.70 to 10.83) |
| 50–59 years | 9.38 (7.19 to 11.31) | 8.59 (5.74 to 10.76) | 8.78 (6.16 to 10.87) |
| >59 years | 9.90 (7.77 to 11.49) | 8.57 (5.62 to 10.81) | 9.20 (6.52 to 11.13) |

**Legend.** IQR = interquartile range.

| Table S7. Coefficient table for dementia models | | | | |
| --- | --- | --- | --- | --- |
|  | **Incident dementia** | | | |
|  | **Overall** | **<50 years** | **50–59 years** | **>59 years** |
| Per 5-point increase in  Brain Care Score | 0.86^***^ | 0.41^***^ | 0.68^***^ | 0.92^***^ |
|  | (0.81 to 0.91) | (0.28 to 0.60) | (0.58 to 0.80) | (0.86 to 0.98) |
| Age | 1.21^***^ |  |  |  |
|  | (1.21 to 1.22) |  |  |  |
| Sex: Males | 1.26^***^ | 1.05 | 1.49^***^ | 1.28^***^ |
|  | (1.19 to 1.33) | (0.69 to 1.60) | (1.26 to 1.75) | (1.20 to 1.35) |
| Observations | 398,753 | 94,347 | 133,657 | 170,749 |
| Cases | 5,354 | 88 | 592 | 4,674 |
| **Legend.** Reference group: females (sex).  ^*^p<0.05; ^**^p<0.01; ^***^p<0.001 | | | | |

| Table S8. Coefficient table for stroke models | | | | |
| --- | --- | --- | --- | --- |
|  | **Incident stroke** | | | |
|  | **Overall** | **<50 years** | **50–59 years** | **>59 years** |
| Per 5-point increase in  Brain Care Score | 0.60^***^ | 0.52^***^ | 0.48^***^ | 0.67^***^ |
|  | (0.57 to 0.63) | (0.44 to 0.61) | (0.44 to 0.53) | (0.63 to 0.71) |
| Age | 1.10^***^ |  |  |  |
|  | (1.10 to 1.10) |  |  |  |
| Sex: males | 1.51^***^ | 1.61^***^ | 1.60^***^ | 1.49^***^ |
|  | (1.44 to 1.58) | (1.36 to 1.92) | (1.45 to 1.76) | (1.41 to 1.57) |
| Observations | 392,789 | 93,817 | 132,086 | 166,886 |
| Cases | 7,259 | 549 | 1,638 | 5,072 |
| **Legend.** Reference group: females (sex). ^*^p<0.05; ^**^p<0.01; ^***^p<0.001 | | | | |

| **Table S9. Coefficient table for composite model** | | | | |
| --- | --- | --- | --- | --- |
|  | **Incident dementia *or* stroke** | | | |
|  | **Overall** | **<50 years** | **50–59 years** | **>59 years** |
| Per 5-point increase in  Brain Care Score | 0.70^***^ | 0.50^***^ | 0.54^***^ | 0.79^***^ |
|  | (0.68 to 0.73) | (0.43 to 0.59) | (0.49 to 0.58) | (0.75 to 0.82) |
| Age | 1.13^***^ |  |  |  |
|  | (1.13 to 1.14) |  |  |  |
| Sex: Males | 1.39^***^ | 1.51^***^ | 1.55^***^ | 1.36^***^ |
|  | (1.34 to 1.44) | (1.29 to 1.78) | (1.42 to 1.69) | (1.31 to 1.42) |
| Observations | 392,574 | 93,807 | 132,041 | 166,726 |
| Cases | 11,705 | 632 | 2,154 | 8,919 |
| **Legend.** Reference group: females (sex). ^*^p<0.05; ^**^p<0.01; ^***^p<0.001 | | | | |

| Table S10. Brain Care Score components, by quintile group | | | | |
| --- | --- | --- | --- | --- |
|  | **1^st^ quintile (n = 73,197)** | **2^nd^–4^th^ quintile (n = 234,208)** | **5^th^ quintile (n = 91,585)** | **Overall (N = 398,990)** |
| **Sex** |  |  |  |  |
| Female | 31,726 (43.3%) | 125,084 (53.4%) | 58,948 (64.4%) | 215,758 (54.1%) |
| Male | 41,471 (56.7%) | 109,124 (46.6%) | 32,637 (35.6%) | 183,232 (45.9%) |
| **Age group** |  |  |  |  |
| <50 years | 14,265 (19.5%) | 49,828 (21.3%) | 30,265 (33.0%) | 94,358 (23.6%) |
| 50–59 years | 26,561 (36.3%) | 77,851 (33.2%) | 29,293 (32.0%) | 133,705 (33.5%) |
| >59 years | 32,371 (44.2%) | 106,529 (45.5%) | 32,027 (35.0%) | 170,927 (42.8%) |
| **Blood pressure** |  |  |  |  |
| 0 | 58,276 (79.6%) | 111,766 (47.7%) | 6,048 (6.6%) | 176,090 (44.1%) |
| 2 | 13,156 (18.0%) | 99,682 (42.6%) | 49,640 (54.2%) | 162,478 (40.7%) |
| 3 | 1,765 (2.4%) | 22,760 (9.7%) | 35,897 (39.2%) | 60,422 (15.1%) |
| Mean (SD) | 0.432 (0.866) | 1.14 (1.13) | 2.26 (0.767) | 1.27 (1.18) |
| **Blood sugar** |  |  |  |  |
| 0 | 7,671 (10.5%) | 7,748 (3.3%) | 621 (0.7%) | 16,040 (4.0%) |
| 1 | 19,519 (26.7%) | 32,783 (14.0%) | 5,356 (5.8%) | 57,658 (14.5%) |
| 2 | 46,007 (62.9%) | 193,677 (82.7%) | 85,608 (93.5%) | 325,292 (81.5%) |
| Mean (SD) | 1.52 (0.678) | 1.79 (0.479) | 1.93 (0.284) | 1.78 (0.505) |
| **Cholesterol** |  |  |  |  |
| 0 | 61,240 (83.7%) | 182,067 (77.7%) | 56,927 (62.2%) | 300,234 (75.2%) |
| 1 | 11,957 (16.3%) | 52,141 (22.3%) | 34,658 (37.8%) | 98,756 (24.8%) |
| Mean (SD) | 0.163 (0.370) | 0.223 (0.416) | 0.378 (0.485) | 0.248 (0.432) |
| **BMI** |  |  |  |  |
| 0 | 35,376 (48.3%) | 53,831 (23.0%) | 4,948 (5.4%) | 94,155 (23.6%) |
| 1 | 29,590 (40.4%) | 113,207 (48.3%) | 29,628 (32.4%) | 172,425 (43.2%) |
| 2 | 8,231 (11.2%) | 67,170 (28.7%) | 57,009 (62.2%) | 132,410 (33.2%) |
| Mean (SD) | 0.629 (0.677) | 1.06 (0.717) | 1.57 (0.594) | 1.10 (0.747) |
| **Nutrition** |  |  |  |  |
| 0 | 42,645 (58.3%) | 82,661 (35.3%) | 15,130 (16.5%) | 140,436 (35.2%) |
| 1 | 25,270 (34.5%) | 104,272 (44.5%) | 40,855 (44.6%) | 170,397 (42.7%) |
| 2 | 5,282 (7.2%) | 47,275 (20.2%) | 35,600 (38.9%) | 88,157 (22.1%) |
| Mean (SD) | 0.490 (0.628) | 0.849 (0.729) | 1.22 (0.710) | 0.869 (0.745) |
| **Alcohol** |  |  |  |  |
| 0 | 46,114 (63.0%) | 109,964 (47.0%) | 23,220 (25.4%) | 179,298 (44.9%) |
| 1 | 20,369 (27.8%) | 86,742 (37.0%) | 40,768 (44.5%) | 147,879 (37.1%) |
| 2 | 6,714 (9.2%) | 37,502 (16.0%) | 27,597 (30.1%) | 71,813 (18.0%) |
| Mean (SD) | 0.462 (0.657) | 0.691 (0.731) | 1.05 (0.743) | 0.731 (0.746) |
| **Smoking** |  |  |  |  |
| 0 | 25,428 (34.7%) | 14,473 (6.2%) | 457 (0.5%) | 40,358 (10.1%) |
| 3 | 47,769 (65.3%) | 219,735 (93.8%) | 91,128 (99.5%) | 358,632 (89.9%) |
| Mean (SD) | 1.96 (1.43) | 2.81 (0.722) | 2.99 (0.211) | 2.70 (0.905) |
| **Aerobic activities** |  |  |  |  |
| 0 | 47,022 (64.2%) | 105,450 (45.0%) | 24,907 (27.2%) | 177,379 (44.5%) |
| 1 | 26,175 (35.8%) | 128,758 (55.0%) | 66,678 (72.8%) | 221,611 (55.5%) |
| Mean (SD) | 0.358 (0.479) | 0.550 (0.498) | 0.728 (0.445) | 0.555 (0.497) |
| **Sleep** |  |  |  |  |
| 0 | 29,601 (40.4%) | 55,071 (23.5%) | 11,158 (12.2%) | 95,830 (24.0%) |
| 1 | 43,596 (59.6%) | 179,137 (76.5%) | 80,427 (87.8%) | 303,160 (76.0%) |
| Mean (SD) | 0.596 (0.491) | 0.765 (0.424) | 0.878 (0.327) | 0.760 (0.427) |
| **Stress** |  |  |  |  |
| 0 | 30,334 (41.4%) | 60,187 (25.7%) | 14,216 (15.5%) | 104,737 (26.3%) |
| 1 | 42,863 (58.6%) | 174,021 (74.3%) | 77,369 (84.5%) | 294,253 (73.7%) |
| Mean (SD) | 0.586 (0.493) | 0.743 (0.437) | 0.845 (0.362) | 0.737 (0.440) |
| **Social relationship** |  |  |  |  |
| 0 | 10,564 (14.4%) | 17,756 (7.6%) | 3,960 (4.3%) | 32,280 (8.1%) |
| 1 | 62,633 (85.6%) | 216,452 (92.4%) | 87,625 (95.7%) | 366,710 (91.9%) |
| Mean (SD) | 0.856 (0.351) | 0.924 (0.265) | 0.957 (0.203) | 0.919 (0.273) |
| **Total Brain Care Score** |  |  |  |  |
| Mean (SD) | 8.05 (1.19) | 11.6 (1.08) | 14.8 (0.951) | 11.7 (2.41) |
| **Legend.** Blood sugar levels were measured as haemoglobin A1c (HbA1C). The red meat score is based on beef, pork, and lamb/mutton consumption, in which an individual score was first assigned for each meat type (“Never” or “Less than once a week” with 0; “Once a week” or “2–4 times a week” with 1; and “5–6 times a week” or “Once or more daily” with 2); these were then summed, with a score of 1–2 dichotomized into 1 and less than 1 or more than 2 with a 0. Moderate activity includes physical activities such as carrying light loads or cycling; vigorous activity includes activities such as fast cycling, aerobics, or heavy lifting. BMI stands for Body Mass Index. | | | | |

The table shows the individual components of the Brain Care Score (number and proportion of participants in each score and the overall mean) by quintile group of the total Brain Care Score. Participants that were categorised into the second, third and fourth quintiles were combined into one group. The total Brain Care Score is the sum of all component scores with a total observed range from 1 to 19.

| Table S11. Hazard Ratios (95%CI) between the Brain Care Score and risk of dementia, stroke, and dementia *or* stroke | | | |
| --- | --- | --- | --- |
|  | **Incident dementia** | **Incident stroke** | **Incident dementia *or* stroke** |
| Per 5-point increase in  Brain Care Score | 0.40^**^ | 0.51^***^ | 0.49^***^ |
|  | (0.23 to 0.71) | (0.39 to 0.66) | (0.39 to 0.61) |
| Age group: 50–59 years | 0.94 | 2.45^*^ | 1.81 |
|  | (0.22 to 4.00) | (1.22 to 4.96) | (0.96 to 3.42) |
| Age group: >59 years | 4.72^*^ | 3.42^***^ | 3.21^***^ |
|  | (1.24 to 18.03) | (1.78 to 6.57) | (1.79 to 5.73) |
| Sex: males | 1.00 | 1.48 | 1.32 |
|  | (0.17 to 5.88) | (0.69 to 3.18) | (0.65 to 2.66) |
| BCS x Age group: 50–59 years | 1.87^*^ | 0.92 | 1.11 |
|  | (1.01 to 3.45) | (0.68 to 1.23) | (0.85 to 1.45) |
| BCS x Age group: >59 years | 2.19^**^ | 1.23 | 1.55^***^ |
|  | (1.24 to 3.87) | (0.94 to 1.62) | (1.2 to 1.98) |
| BCS x Sex: males | 1.02 | 1.04 | 1.06 |
|  | (0.47 to 2.24) | (0.75 to 1.44) | (0.79 to 1.44) |
| Age group: 50-59 years x Sex: males | 2.28 | 0.95 | 1.22 |
|  | (0.33 to 15.62) | (0.39 to 2.28) | (0.55 to 2.71) |
| Age group: >59 years x Sex: males | 1.07 | 0.78 | 0.88 |
|  | (0.18 to 6.45) | (0.34 to 1.75) | (0.42 to 1.84) |
| BCS x Age group: 50–59 years x Sex: males | 0.81 | 1.02 | 0.93 |
|  | (0.34 to 1.89) | (0.70 to 1.50) | (0.65 to 1.31) |
| BCS x Age group: >59 years x Sex: males | 1.05 | 1.08 | 1.01 |
|  | (0.48 to 2.34) | (0.76 to 1.53) | (0.74 to 1.39) |
| Observations | 398,753 | 392,789 | 392,574 |
| Cases | 5,354 | 7,259 | 11,705 |
| **Legend.** BCS = Per 5-point increase in Brain Care Score; reference groups: >50 years (age group), females (sex). ^*^p<0.1; ^**^p<0.05; ^***^p<0.01 | | | |

The table shows the Hazard Ratios and corresponding 95% Confidence Intervals of associations between the Brain Care Score and risk of dementia, stroke, and dementia *or* stroke featuring two- and three-way interactions between the BCS, age groups, and sex.

| Table S12. Cumulative incidence of dementia, stroke, and dementia or stroke at baseline by Brain Care Score quintile group | | | |
| --- | --- | --- | --- |
|  | **Cumulative incidence of dementia (95% CI)** | **Cumulative incidence of stroke (95% CI)** | **Cumulative incidence of the composite outcome (95% CI)** |
| Overall | 1.3% (1.3% to 1.4%) | 1.8% (1.8% to 1.9%) | 3.0% (2.9% to 3.0%) |
| 1^st^ quintile (BCS: 1–9) | 1.5% (1.5% to 1.6%) | 2.7% (2.6% to 2.8%) | 3.9% (3.8% to 4.1%) |
| 2^nd^–4^th^ quintile (BCS: 10–13) | 1.4% (1.3% to 1.4%) | 1.95 (1.8% to 1.9%) | 3.0% (3.0% to 3.1%) |
| 5^th^ quintile (BCS: 14–19) | 1.1% (1.0% to 1.1%) | 1.2% (1.1% to 1.3%) | 2.1% (2.0% to 2.2%) |

| **Legend.** BCS = Brain Care Score; 95% CI = 95% confidence interval. |
| --- |

| Table S13. Coefficient table for dementia Fine and Gray subdistribution hazard model | | | | |
| --- | --- | --- | --- | --- |
|  | **Incident dementia** | | | |
|  | **Overall** | **<50 years** | **50–59 years** | **>59 years** |
| Per 5-point increase in  Brain Care Score | 0.89^***^ | 0.41^***^ | 0.69^***^ | 0.95 |
|  | (0.84 to 0.95) | (0.27 to 0.63) | (0.58 to 0.83) | (0.89 to 1.01) |
| Age | 1.21^***^ |  |  |  |
|  | (1.20 to 1.21) |  |  |  |
| Sex: males | 1.21^***^ | 1.05 | 1.47^***^ | 1.23^***^ |
|  | (1.14 to 1.27) | (0.68 to 1.61) | (1.25 to 1.73) | (1.16 to 1.30) |
| Observations | 398,753 | 94,347 | 133,657 | 170,749 |
| **Legend.** Reference group: females (sex). ^*^p<0.05; ^**^p<0.01; ^***^p<0.001 | | | | |

| Table S14. Coefficient table for stroke Fine and Gray subdistribution hazard model | | | | |
| --- | --- | --- | --- | --- |
|  | **Incident stroke** | | | |
|  | **Overall** | **<50 years** | **50–59 years** | **>59 years** |
| Per 5-point increase in  Brain Care Score | 0.62^***^ | 0.53^***^ | 0.49^***^ | 0.69^***^ |
|  | (0.59 to 0.65) | (0.44 to 0.62) | (0.45 to 0.55) | (0.65 to 0.74) |
| Age | 1.10^***^ |  |  |  |
|  | (1.09 to 1.10) |  |  |  |
| Sex: males | 1.47^***^ | 1.61^***^ | 1.58^***^ | 1.44^***^ |
|  | (1.40 to 1.54) | (1.35 to 1.92) | (1.43 to 1.75) | (1.36 to 1.52) |
| Observations | 392,789 | 93,817 | 132,086 | 166,886 |
| **Legend.** Reference group: females (sex). ^*^p<0.05; ^**^p<0.01; ^***^p<0.001 | | | | |

| Table S15. Coefficient table for composite Fine and Gray subdistribution hazard model | | | | |
| --- | --- | --- | --- | --- |
|  | **Incident dementia *or* stroke** | | | |
|  | **Overall** | **<50 years** | **50–59 years** | **>59 years** |
| Per 5-point increase in  Brain Care Score | 0.72^***^ | 0.51^***^ | 0.55^***^ | 0.81^**^ |
|  | (0.70 to 0.75) | (0.43 to 0.60) | (0.50 to 0.60) | (0.77 to 0.85) |
| Age | 1.13^***^ |  |  |  |
|  | (1.12 to 1.13) |  |  |  |
| Sex: males | 1.35^***^ | 1.51^***^ | 1.53^***^ | 1.32^***^ |
|  | (1.30 to 1.40) | (1.28 to 1.77) | (1.41 to 1.67) | (1.27 to 1.38) |
| Observations | 392,574 | 93,807 | 132,041 | 166,726 |
| **Legend.** Reference group: females (sex). ^*^p<0.05; ^**^p<0.01; ^***^p<0.001 | | | | |

Fig. S1 Schoenfeld residuals for proportional hazards assumption with incident dementia as outcome **a) Main model**

**b) <50 years**

**c) 50–59 years**

**d) >59 years**

# Fig. S2 Schoenfeld residuals for proportional hazards assumption with incident stroke as outcome

**a) Main model**

**b) <50 years**

**c) 50–59 years**

**d) >59 years**

# Fig. S3 Schoenfeld residuals for proportional hazards assumption with incident dementia *or* stroke as outcome

**a) Main model**

**b) <50 years**

**c) 50–59 years**

**d) >59 years**
